# Correlation between arterial spin labeling MRI and dynamic FDG on PET-MR in Alzheimer’s disease and non-Alzhiemer’s disease patients

**DOI:** 10.1186/2197-7364-2-S1-A83

**Published:** 2015-05-18

**Authors:** David Douglas, Maged Goubran, Eugene Wilson, Guofan Xu, Pragya Tripathi, Dawn Holley, Steven Chao, Max Wintermark, Andrew Quon, Michael Zeineh, Minal Vasanawala, Greg Zaharchuk

**Affiliations:** Stanford University, California, USA

Regional hypoperfusion on Arterial Spin Labeling (ASL) MRI and corresponding regions of hypometabolism on FDG PET have been reported in Alzheimer’s Disease (AD). To our knowledge these correlations have not been studied under simultaneous acquisition. The purpose of this study is to investigate the correlation of ASL with FDG PET under simultaneous acquisition on PET-MR and to explore this correlation as a possible biomarker for AD. Dynamic FDG and ASL imaging was performed using a simultaneous TOF-enabled PET-MR scanner in 7 subjects without AD and 3 subjects with AD. Average age was 68±5 years. Automated atlas-based segmentation was performed using T2 MRI using the Talairach atlas. Quantitative analysis of ASL and FDG (delayed 45-75 minute scan) was performed in five regions using the pons as a reference region for both perfusion and metabolism. Statistical analyses included Spearman’s correlation and student’s t-test. Significant correlation of relative perfusion and metabolism was found in two of the five brain regions including the putamen (p = 0.018) and the hippocampus (p = 0.031). In addition, there was significant difference between the relative perfusion and metabolism of the thalamus (p = 0.04). No difference was seen between the AD and non-AD groups. Simultaneous PET-MR demonstrates a positive correlation of perfusion of ASL with metabolism on FDG PET in the hippocampus and putamen. The putamen correlation has previously been reported in the literature on a non-simultaneous ASL and FDG imaging. The thalamus was noted to have a difference in the relative perfusion and metabolism representing a perfusion-metabolism mismatch. Future studies should explore the correlation in additional brain regions and the meaning of perfusion-metabolism mismatches as potential imaging biomarkers for patients with and without AD.

